# Effects of intraperitoneal injection of microencapsulated Sertoli cells on chronic and presymptomatic dystrophic mice

**DOI:** 10.1016/j.dib.2015.11.016

**Published:** 2015-11-15

**Authors:** Sara Chiappalupi, Giovanni Luca, Francesca Mancuso, Luca Madaro, Francesca Fallarino, Carmine Nicoletti, Mario Calvitti, Iva Arato, Giulia Falabella, Laura Salvadori, Antonio Di Meo, Antonello Bufalari, Stefano Giovagnoli, Riccardo Calafiore, Rosario Donato, Guglielmo Sorci

**Affiliations:** a Department of Experimental Medicine, University of Perugia, Perugia 06132, Italy; b Department of Medicine, University of Perugia, Perugia, Italy; c IRCCS Fondazione Santa Lucia, Rome 00143, Italy; d National Research Council, Institute of Cell Biology and Neurobiology, Fondazione Santa Lucia, Rome 00143, Italy; e Unit of Histology, DAHFMO, La Sapienza University, Rome 00161, Italy; f Department of Veterinary Medicine, University of Perugia, Perugia 06126, Italy; g Department of Pharmaceutical Sciences, University of Perugia, Perugia 06123, Italy; h Interuniversity Institute of Myology (IIM), Italy

## Abstract

We report data about the effects of intraperitoneal (i.p.) injection of specific pathogen-free (SPF) porcine Sertoli cells (SeC) encapsulated into clinical grade alginate-based microcapsules (SeC-MC) on muscles of chronic and presymptomatic dystrophic, *mdx* mice. *Mdx* mouse is the best characterized animal model of Duchenne muscular dystrophy (DMD), an X-linked lethal myopathy due to mutation in the gene of dystrophin, which is crucial for myofiber integrity during muscle contraction. Our data show that three weeks after i.p. injection of SeC-MC significantly reduced adipose and fibrous tissue deposition, reduced macrophage infiltrate, and reduced numbers of damaged myofibers are found in muscles of 12-month-old *mdx* mice, which reproduce chronic DMD conditions. Compared with muscles of mock-treated *mdx* mice muscles of SeC-MC-treated mice show upregulation of the dystrophin paralogue, utrophin which is localized to the periphery of myofibers. Moreover, our data show that i.p. injection of SeC-MC into presymptomatic, 2-week-old *mdx* mice, although not fully preventing myofiber degeneration, results in protection against myofiber necrosis and muscle inflammation. Extensive discussion of these data can be found in Ref. [1].

**Specifications Table**

**Table:** 

Subject area	Biology
More specific subject area	Translational medicine/Preclinical studies
Type of data	Text file and figures
How data was acquired	Bright field microscope Olympus BX51; epifluorescence microscope Leica DMRB; C-DiGit Blot Scanner (LI-COR)
Data format	Raw; analyzed
Experimental factors	Intraperitoneal injection of microencapsulated SPF porcine Sertoli cells (SeC-MC) in chronic and presymptomatic dystrophic (mdx) mice. Three weeks after injection, muscles of mock- and SeC-MC-treated mdx mice were analyzed for several parameters.
Experimental features	Muscle tissue were formalin-fixed paraffin embedded or fresh-frozen for histology, immunohistochemistry, immunofluorescence or Evans blue dye (EBD) infiltration tests, or were homogenized for western blotting analysis.
Data source location	University of Perugia, Perugia 06132, Italy; IRCCS Fondazione Santa Lucia, Rome 00143, Italy; La Sapienza University, Rome 00161, Italy
Data accessibility	The data are supplied with this article

**Value of the data**

Inflammatory events secondary to lack of dystrophin play a major role in the outcome of DMD pathology [2], [3], so that antiinflammatory steroids represents the current standard treatment for DMD patients, albeit with limited efficacy and undesired side effects [4], [5], [6].

The therapeutic approaches to DMD proposed so far have revealed intrinsic limitations and/or require pharmacological immunosuppression, so that combinatorial approaches are encouraged [4].

Here we propose a therapeutic approach that combines the antiinflammatory and immunomodulatory properties of Sertoli cells (SeC) with the safety of using microencapsulated cells. We show that inside the microcapsules SeC act as a “micro-biofactory” and drug delivery system able to improve muscle morphology and performance by secreting immunomodulatory and trophic factors [7], [8], [9] once injected into the peritoneal cavity of dystrophic mice.

Our data open new perspective in the treatment of DMD and related myopathies.

## 1 Data

**Fig. 1:**
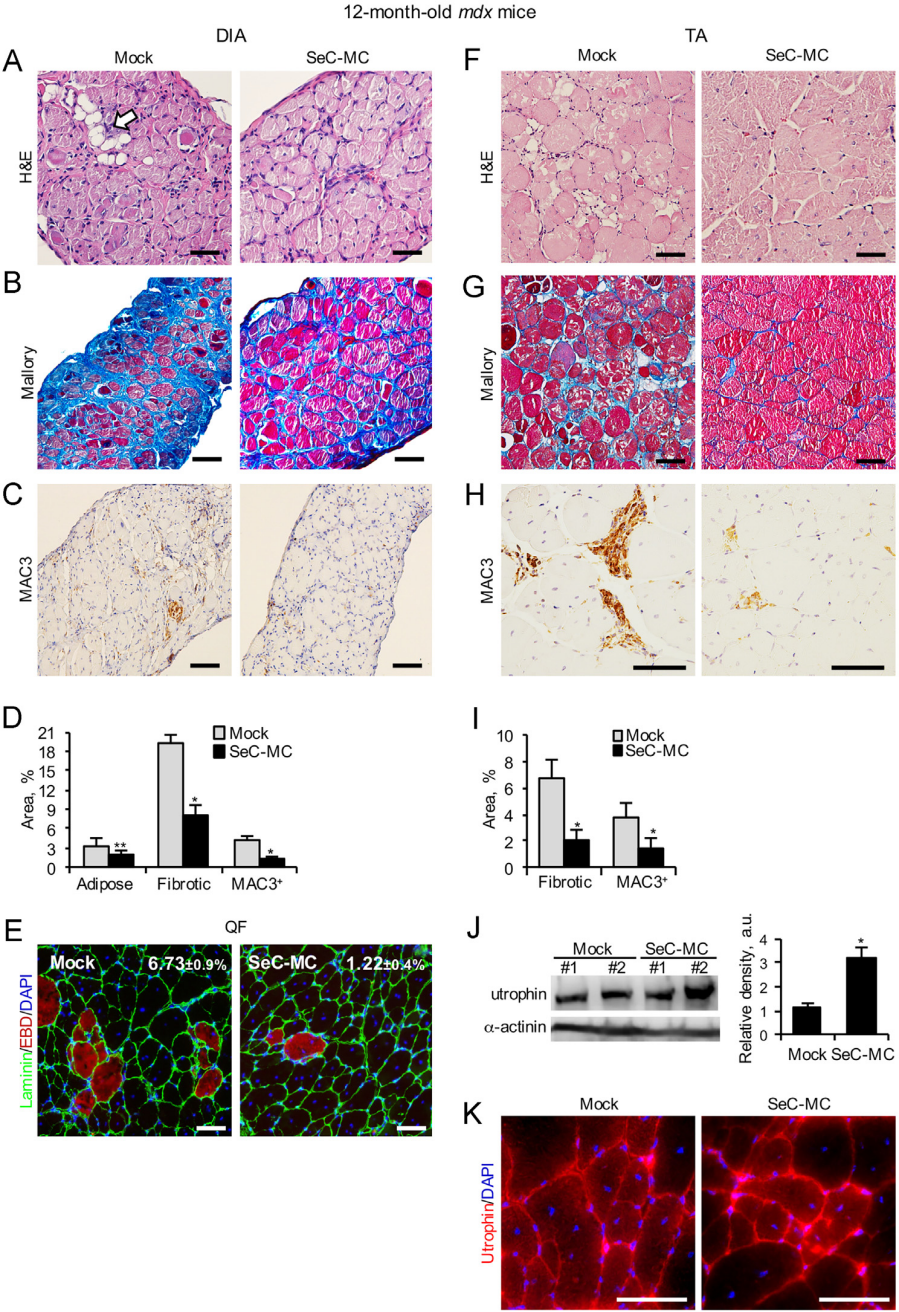
SeC-MC ameliorate muscle morphology when i.p. injected into chronic dystrophic mice. (A–D) DIA from 12-month-old *mdx* mice i.p. injected with SeC-MC (*n*=6) or empty microcapsules (Mock) (*n*=6) were analyzed three weeks after injection. H&E (A) and Mallory (B) staining were used to reveal adipose tissue (*arrow* in A) and fibrotic tissue (*blue*) infiltrate, respectively, and MAC3 immunohistochemistry (C) was used to detect macrophages in DIA from SeC-MC-treated and mock-treated mice. The average percentages (±SEM) of adipose tissue infiltrate, and fibrotic and MAC3^+^ areas in mock- and SeC-MC-treated DIA were determined (D). (E) *Mdx* mice treated as above were injected with Evans Blue Dye (EBD) 24 h before being sacrificed and QF muscles were isolated, cryosectioned and analyzed for detection of EBD infiltration (*red*). Individual myofibers were delineated with laminin staining (*green*), and nuclei were counterstained with DAPI (*blue*). Shown are representative images. Reported are the average percentages (±SEM) of EBD-positive myofibers in each group. (F–K) TA muscles isolated from mice in (A–D) were analyzed by H&E (F) or Mallory (G) staining, and MAC3 immunohistochemistry (H). The mean percentages (±SEM) of fibrotic and MAC3^+^ areas in mock- and SeC-MC-treated mice were determined (I). Utrophin expression and localization were analyzed by Western blotting (J) and immunofluorescence on frozen sections (K), respectively. The average relative densities (±SEM) of utrophin bands with respect to α-actinin bands were determined (J). * and **, significantly different from mock-treated control at *p*≤0.001 and *p*≤0.01, respectively. Original magnification (A–C, E–G), 20×; (H and K), 40×.

We injected microencapsulated Sertoli cells (SeC-MC) or empty microcapsules (E-MC) into the peritoneal cavity of 12-month-old *mdx* mice, which reproduce chronic DMD conditions since their muscles (especially the diaphragms, DIA) progressively accumulate fibrous and adipose tissue [10]. Three weeks after injection, SeC-MC induced a significant reduction of fat and fibrous tissue deposition in muscles (Fig. 1A, B, D, G and I). Moreover, SeC-MC treatment resulted in reduced macrophage infiltrate and muscle damage, as investigated by MAC3 immunohistochemistry in DIA and *Tibialis anterior* (TA) muscles (Fig. 1C, D, H and I), and autofluorescence analysis of the *Quadriceps femoris* (QF) after Evans blue dye (EBD) injection (Fig. 1E). Compared with muscles of mock-treated *mdx* mice, muscles of SeC-MC-treated *mdx* mice showed upregulated utrophin (3.2-fold increase in protein amount) (Fig. 1J), which was localized to the periphery of the myofibers (Fig. 1K), a condition necessary for utrophin to functionally replace dystrophin.

**Fig. 2:**
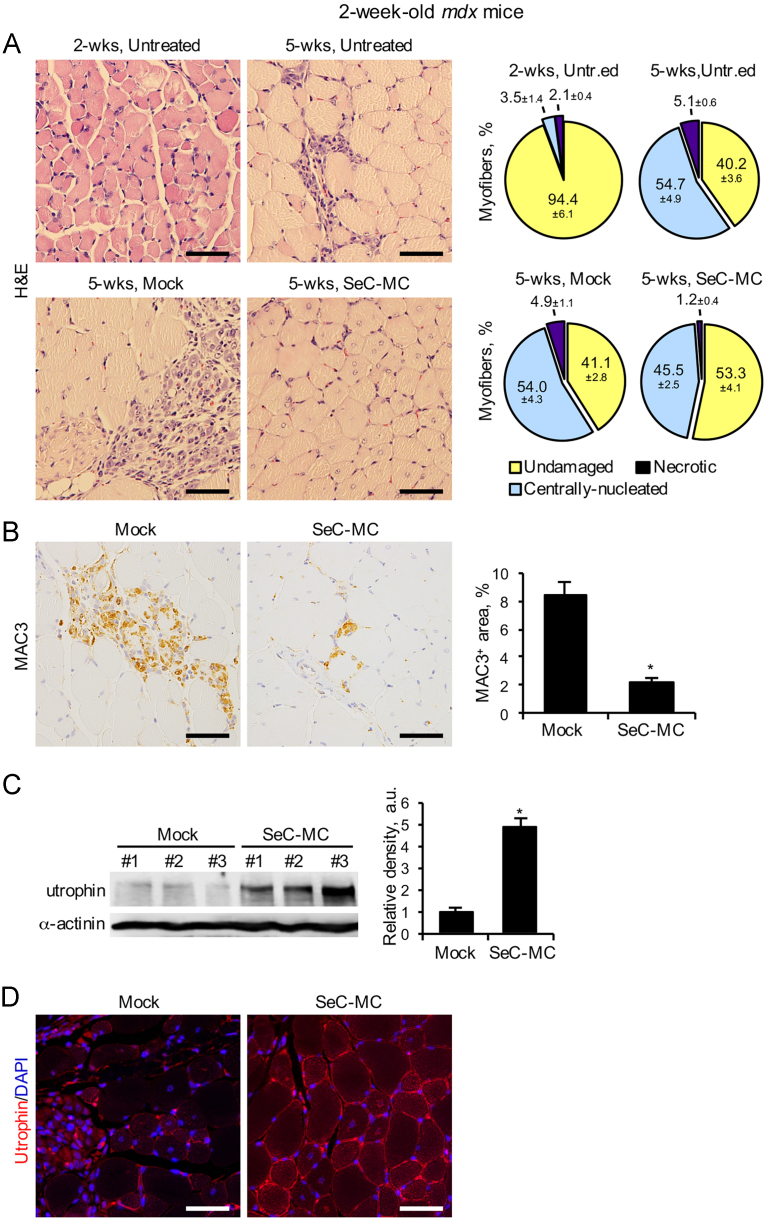
SeC-MC reduce muscle necrosis when i.p. injected into presymptomatic dystrophic mice. (A) Two-week-old *mdx* mice were i.p. injected with E-MC (mock) (*n*=6) or SeC-MC (*n*=6). Three weeks after injection TA muscle morphology was analyzed by haematoxylin/eosin staining (*lower left panels*) and the mean percentages (±SEM) of undamaged, centrally-nucleated, and necrotic myofibers were determined (*lower right panels*).TA muscles of untreated 2-week- and 5-week-old *mdx* mice (*n*=5 each group) were analyzed in parallel (*upper panels*). (B) TA muscles from mock- and SeC-MC-treated *mdx* mice in (A) were analyzed for infiltrating macrophages by MAC3 immunohistochemistry (*brown*). The mean percentages (±SEM) of MAC3^+^ areas were determined. (C) Utrophin expression was analyzed by Western blotting. The average relative densities (±SEM) of utrophin bands with respect to α-actinin bands were determined. (D) Utrophin localization was determined by immunofluorescence on formalin-fixed paraffin-embedded muscle tissue. Nuclei were counterstained with DAPI (*blue*). Shown are representative images. *, significantly different from control (*p*≤0.001). Original magnification (A, B, and D), 40×.

Treatment with SeC-MC in pre-symptomatic 2-week-old *mdx* mice, which show only minor signs of muscle degeneration (94.4±6.1% undamaged myofibers in TA muscles) (Fig. 2A) resulted in not full prevention but protection against the necrosis of myofibers and muscle inflammation, as evidenced by histological and immunohistochemical analyses performed three weeks after injection (Fig. 2A and B). Also in this case, utrophin expression resulted increased (about five-fold increase compared to mock-treated animals) and localized at the periphery of myofibers (Fig. 2C and D). Incidentally, our data show that pathogenic mechanisms leading to muscle degeneration and subsequent activation of regeneration cycles are already active in *mdx* mice at the age of two weeks, as evidenced by histological analysis of the percentages of centrally-nucleated myofibers in SeC-MC-treated mice at the beginning and the end of the treatment (Fig. 2A).

## 2 Experimental design, materials and methods

### 2.1 Microencapsulation of SeC

The procedures for the isolation of Sertoli cells (SeC) from SPF-certified pre-pubertal Large White pig testes, and SeC characterization and microencapsulation into sodium alginate are fully described in Ref. [1].

### 2.2 Animals

Data were obtained in 2-week- or 12-month-old male *mdx* mice (C57BL/10ScSn-*Dmd*^*mdx*^/J, original breeding from Jackson Laboratory) raised on a 12 h light/day cycle and a standard mouse diet. SeC-MC or E-MC were injected into the peritoneal cavity of recipient mice by a sterile 16-gauge catheter under general anesthesia. Equivalent amounts to 1.0×10^6^ SeC/g body weight in a concentration of 1.0×10^7^ SeC/ml in saline (NaCl 0.9%) were transplanted. The procedures and experiments on mice were approved by the Ethics Committee of the Perugia University and the Italian Ministry of Health.

### 2.3 Morphological analyses

Muscles were isolated, formalin-fixed and paraffin-embedded in order to maximally preserve morphology. Muscle cross-sections measuring 4 µm were obtained and processed for standard haematoxylin/eosin (H&E) or Mallory staining. Quantifications on muscle sections were performed by three independent operators blinded to treatments. Sections at 100 µm intervals for each muscle were analyzed. The entire area of each section was evaluated to avoid bias. Areas with sectioning artifacts (folds, tears, etc.) were avoided. After H&E staining, the percentages of undamaged, regenerating, regenerated, and necrotic myofibers per section were manually counted by three independent operators according to the following parameters: i) undamaged fibers, identified by the presence of peripheral nuclei; ii) regenerated fibers, identified by normal size but with central nuclei; iii) regenerating fibers, identified by small size, basophilic cytoplasm and central nuclei; and, iv) necrotic fibers, identified by pale cytoplasm and phagocytosis. Areas of fat deposition were identified by the evacuated spaces generated by the histochemical delipidation. The percentages of adipose tissue and fibrotic areas (*blue*, after Mallory staining) were measured using Scion Image 4.0.3.2 software (NIH, Bethesda, USA). Slices were analyzed and photographed with a bright field microscope (Olympus BX51) equipped with a digital camera. Values reported are given as mean (±SEM) obtained from multiple animals as specified in each figure. For the evaluation of myofiber necrosis mice were i.p. injected with a 1% Evan׳s Blue Dye (EBD) solution (Sigma-Aldrich) at 1% volume relative to body mass 16–24 h prior to tissue sampling. Muscles were isolated and flash frozen in a pre-cooled beaker of isopentane placed in liquid nitrogen. Cryosections were fixed in acetone at −20°C. An anti-laminin antibody (Sigma-Aldrich, L9393; 1:50) followed by an AlexaFluor 488-conjugated anti-rabbit Ig G (Invitrogen, 1:100) was used to mark individual myofibers. Nuclei were counterstained with 4′, 6-diamidino-2-phenylindole (DAPI; Sigma-Aldrich). Negative controls (not shown) bypassed the primary antibody treatment. The samples were analyzed by an epifluorescence microscope (Leica DMRB) equipped with a digital camera.

### 2.4 Immunohistochemistry and immunofluorescence

Paraffin sections of muscles were cut at 4 µm, deparaffinized with xylene and rehydrated in a graded ethanol series. Antigen retrieval was obtained by boiling for 1.5 h in 10 mM citric acid buffer (pH 6.0), and depletion of endogenous peroxidase was accomplished by treatment with 3% H_2_O_2_. Sections were washed with TBS, pH 7.4, incubated for 1 h with blocking buffer [BB; TBS containing 0.01% Tween-20 (T-TBS) and 10% HS]. MAC3 detection was obtained by an anti-MAC3 antibody (BD Biosciences,1:50 in BB) followed by incubation with horseradish peroxidase (HRP)-conjugated anti-rat Ig G antibody (Santa Cruz Biotechnology, 1:500). Sections were incubated with 0.01% 3,3-diaminobenzidine tetrahydrochloride (DAB), and 0.006% H_2_O_2_ in 50 mM Tris–HCl, pH 7.4. Nuclei were counterstained with haematoxylin. The slices were then dehydrated, mounted with EuKitt mounting medium (Electron Microscopy Sciences, USA), and analyzed and photographed with a bright field microscope (Olympus BX51) equipped with a digital camera. To detect utrophin in muscle tissue (Fig. 2D), immunofluorescence reactions on tissue slices were performed as above except that PBS, pH 7.4, and a different BB (i.e., 0.4% Triton-X-100, 10% donkey serum and 1% BSA in PBS) were used. A mouse monoclonal anti-utrophin (8A4) antibody (Santa Cruz Biotechnology, sc-33700; 1:20 in BB) was used as primary antibody, followed by a TRITC-conjugated anti-mouse IgG antibody (Sigma-Aldrich, 1:50). Where indicated, acetone-fixed cryosections (7 µm) were used to detect utrophin using the same primary and secondary antibodies as above diluted in 1% BSA in PBS. Nuclei were counterstained with DAPI. After rinsing, samples were mounted with fluorescent mounting medium (Dako Corporation, Denmark) and viewed in an epifluorescence microscope (Leica DMRB) equipped with a digital camera.

## 3 Western blotting

Muscle tissue was homogenized in 10 mM Tris, pH 7.4, 2.5% v/v sodium dodecyl sulfate (SDS), 100 mM dithiothreitol (DTT), in the presence of a mixture of protease inhibitors (Sigma-Aldrich). The amount of protein in each sample was determined by Bradford assay, and equal amounts of protein were size-separated by SDS–PAGE. The following primary antibodies were used: anti-utrophin (8A4) (Santa Cruz Biotechnology, sc-33700; 1:500); anti-α-actinin (Sigma-Aldrich, cat. A7811; 1:1000). After incubation with the appropriate HRP-conjugated secondary antibodies, the immune reaction was developed by enhanced chemiluminescence (SuperSignal West Pico, Thermo Scientific). C-DiGit Blot Scanner (LI-COR, USA) was used for the analysis of blots.

## 4 Statistical analysis

Quantitative data are means±SEM of at least three independent experiments. Representative experiments are shown unless stated otherwise. The data were subjected to analysis of variance (ANOVA) with SNK post-hoc analysis using a statistical software package (GraphPad Prism version 4.00, GraphPad).

## References

[1] Chiappalupi S., Luca G., Mancuso F., Madaro L., Fallarino F., Nicoletti C., Calvitti M., Arato I., Falabella G., Salvadori L., Di Meo A., Bufalari A., Giovagnoli S., Calafiore R., Donato R., Sorci G. (2016). Intraperitoneal injection of microencapsulated Sertoli cells restores muscle morphology and performance in dystrophic mice. Biomaterials.

[2] Porter J.D., Khanna S., Kaminski H.J., Rao J.S., Merriam A.P., Richmonds C.R., Leahy P., Li J., Guo W., Andrade F.H. (2002). A chronic inflammatory response dominates the skeletal muscle molecular signature in dystrophin-deficient mdx mice. Hum. Mol. Genet..

[3] Evans N.P., Misyak S.A., Robertson J.L., Bassaganya-Riera J., Grange R.W. (2009). Immune mediated mechanisms potentially regulate the disease time course of Duchenne muscular dystrophy and provide targets for therapeutic intervention. PM&R.

[4] Khurana T.S., Davies K.E. (2003). Pharmacological strategies for muscular dystrophy. Nat. Rev. Drug Discov..

[5] Goyenvalle A., Seto J.T., Davies K.E., Chamberlain J. (2011). Therapeutic approaches to muscular dystrophy. Hum. Mol. Genet..

[6] Manzur A.Y., Kuntzer T., Pike M., Swan A.V. (2008). Glucocorticoid corticosteroids for Duchenne muscular dystrophy. Cochrane Database Syst. Rev..

[7] Skinner M.K., Griswold M.D. (2005). Sertoli Cell Biology.

[8] Mital P., Kaur G., Dufour J.M. (2010). Immunoprotective Sertoli cells: making allogeneic and xenogeneic transplantation feasible. Reproduction.

[9] Shamekh R., El-Badri N.S., Saporta S., Pascual C., Sanberg P.R., Cameron D.F. (2006). Sertoli cells induce systemic donor-specific tolerance in xenogenic transplantation model. Cell Transpl..

[10] Stedman H.H., Sweeney H.L., Shrager J.B., Maguire H.C., Panettieri R.A., Petrof B., Narusawa M., Leferovich J.M., Sladky J.T., Kelly A.M. (1991). The mdx mouse diaphragm reproduces the degenerative changes of Duchenne muscular dystrophy. Nature.

